# A Novel Variant in *NR5A1* Presenting as 46,XY Difference of Sex Development

**DOI:** 10.1210/jcemcr/luad103

**Published:** 2023-09-15

**Authors:** Yunting Yu, Peter A Lee, Lina Huerta-Saenz, Natalie G Allen

**Affiliations:** Penn State College of Medicine, Hershey, PA 17033, USA; Penn State College of Medicine, Hershey, PA 17033, USA; Department of Pediatrics, Division of Pediatric Endocrinology and Diabetes, Penn State Health Milton S. Hershey Medical Center, Hershey, PA 17033, USA; Penn State College of Medicine, Hershey, PA 17033, USA; Department of Pediatrics, Division of Pediatric Endocrinology and Diabetes, Penn State Health Milton S. Hershey Medical Center, Hershey, PA 17033, USA; Penn State College of Medicine, Hershey, PA 17033, USA; Department of Pediatrics, Division of Pediatric Endocrinology and Diabetes, Penn State Health Milton S. Hershey Medical Center, Hershey, PA 17033, USA

**Keywords:** differences of sex development, ambiguous genitalia

## Abstract

Differences of sex development (DSDs) are a spectrum of congenital clinical conditions involving the development of gonadal, chromosomal, and anatomical sex. The physical presentation provides incomplete clues because underlying etiologies may present with similar findings. We describe an 8-year-old boy from the Dominican Republic originally diagnosed with congenital adrenal hyperplasia (CAH). He was prescribed oral hydrocortisone and fludrocortisone, with irregular adherence. During infancy, he had human chorionic gonadotropin injections to stimulate phallic growth. After migrating to the United States, medications became depleted but without adrenal crisis. Laboratory testing with high-dose adrenocorticotropin stimulation study ruled out CAH. Careful examination noted an underdeveloped bifid scrotum, bilaterally undescended testicles, a 2-cm phallus, severe penoscrotal hypospadias, and chordee. Subsequently, he had a 2-stage bilateral orchiopexy and surgical repair of penoscrotal hypospadias and chordee. Genetic testing for 46,XY DSD revealed a novel, dominant, heterozygous, likely pathogenic variant (c.102 + 1G > C) in the *NR5A1* gene associated with severe phenotype of undervirilized male. This case illustrates the crucial role of molecular genetic testing for the diagnosis of 46,XY DSDs and a novel *NR5A1* gene variant.

## Introduction

Differences of sex development (DSDs) are a spectrum of congenital clinical conditions affecting the development of gonadal, chromosomal, or anatomical sex [1]. Broad categories of DSD include 46,XX; 46,XY; and sex chromosome DSDs. The most common diagnosis is gonadal dysgenesis in 46,XY DSDs and congenital adrenal hyperplasia (CAH) in those classified as 46, XX DSD.

The *NR5A1* gene located on chromosome 9q33.3 encodes steroidogenic factor-1 (SF-1), a key transcription factor involved in the regulation of adrenal and gonadal development and function. Original studies of SF-1 knockout mice revealed severe adrenocortical insufficiency from lack of adrenal glands and gonadal agenesis, suggesting the essential role of SF-1 in the development of steroidogenic organs [2].

In humans, mutations of SF-1 have been hypothesized to affect sex-determining-region Y (*SRY*) and SRY-box 9 (*SOX9*), leading to phenotypic cases of 46,XY DSD. *NR5A1* has also been suggested to play a role in the inhibition of pro-ovarian pathways toward male development [3]. To date, 46,XY patients with *NR5A1* mutations have shown a wide range of clinical phenotypes of partial gonadal dysgenesis, cryptorchidism, micropenis, lack of müllerian remnants, and hypospadias [4-6]. Here we present the case of an 8-year-old boy from the Dominican Republic.

## Case Presentation

In 2018, an 8-year-old Spanish-speaking boy from the Dominican Republic with a history of CAH and ambiguous genitalia was referred for medical care. The patient, the only living child of his biological parents, was born at full-term after his mother had 2 spontaneous miscarriages. He had a younger maternal half-brother. The mother denied consanguinity, polycystic ovary syndrome, and hormone use during pregnancy. In the Dominican Republic, he had human chorionic gonadotropin injections for microphallus treatment as a neonate. Later, he was diagnosed with CAH with medical reasoning and testing completed not available to our team. He was taking hydrocortisone 7 mg/m^2^/day and fludrocortisone 0.1 mg oral daily the year he moved to the United States. Although adherence was poor according to his family, he had no adverse effects or evidence of mineralocorticoid or glucocorticoid insufficiency. He denied pain or difficulties when urinating; however, he urinated in a sitting position. He identified as a male and frequently asked why his genitals looked different from those of his younger brother.

After immigrating to the United States, he was without medications for 3 months. He presented to a local emergency room complaining of fatigue and nonreproducible periumbilical pain, raising concern for adrenal crisis. On presentation, his vital signs and serum laboratory values, including electrolytes, were within normal limits. He was afebrile (37.3 °C/99.1 °F) and normotensive (114/73 mm Hg). He had had one episode of fever to 38.3 °C/101 °F, but denied nausea, vomiting, syncope, shortness of breath, or other symptoms. This presentation was inconsistent with adrenal crisis.

## Diagnostic Assessment

On initial presentation to the endocrine clinic, the patient was noted to have bilaterally undescended testes, a bifid scrotum and penoscrotal hypospadias, associated with significant ventral penile curvature with perineal opening only on the ventral side of the phallus. Stretched penile length was 2 cm, and he was Tanner stage 1 for pubic hair. Based on physical examination, the External Masculinization Score (range 0-12) was 3 and the Prader score was 4.

Initial imaging with ultrasound and magnetic resonance imaging identified an unremarkable left adrenal gland, no visualization of the right adrenal gland, and no uterus or ovaries. Pelvic magnetic resonance imaging scan also showed rudimentary testes in the inguinal canal bilaterally. Testicular measurements were 2.8 × 1.1 cm on the right and 2.2 × 1 cm on the left.

Diagnostic workup included normal electrolytes, 17-hydroxyprogesterone (17-OHP) of 20 ng/dL (normal reference [NR]: <91 ng/dL); less than 0.01 nmol/L (NR: <2.75 nmol/L), and adrenocorticotropin stimulation test showing stimulated 17-OHP of 100 ng/dL (NR: <335 ng/dL); 0.03 nmol/L (NR: <10.14 nmol/L); and cortisol level of 19.6 μg/dL (NR: >18 μg/dL); 540 nmol/L (NR: >497 nmol/L), inconsistent with CAH.

Luteinizing hormone (LH) and follicle-stimulating hormone (FSH) were low, consistent with a prepubertal child, as there was an undetectable total testosterone level (Table 1). Other laboratory results, including dihydrotestosterone and dehydroepiandrosterone sulfate, were normal. Karyotype was 46,XY and genetic testing for an *SRD5A2* mutation for 5-alpha reductase was negative. Subsequent laboratory test results suggested low Sertoli cell function including low inhibin B and low antimüllerian hormone (AMH) (see Table 1).

**Table 1.** Laboratory values and physical examination measurements from initial presentation in endocrine clinic to most recent visit^**^

	03/2018 Age 8	07/2018 Age 8	08/2018 Age 8	01/2019 Age 9	03/2020 Age 10	02/2021^*^ Age 10	08/2021 Age 11	02/2023 Age 13
**LH (mIU/mL; IU/L)**	**<0.20; <0.20** ***(NR: 0.02*-*0.30; 0.02*-*0.30)***	**0.30, 0.30** ***(NR: 0.02*-*0.30; 0.02*-*0.30)***	**0.40; 0.40** ***(NR: 0.02*-*0.30; 0.02*-*0.30)***	**0.60; 0.60** ***(NR: 0.02*-*0.30; 0.02*-*0.30)***	**0.60; 0.60** ***(NR: 0.2*-*4.90; 0.2*-*4.90)***	**4.40; 4.40** ***(NR: 0.2*-*5.0; 0.2*-*5.0)***		**25.00; 25.00** ***(NR: 0.4*-*7.0; 0.4*-*7.0)***
**FSH (mIU/mL; IU/L)**	**0.8; 0.8** ***(NR: 0.26*-*3.0; 0.26*-*3.0)***	**2.90; 2.90** ***(NR: 0.26*-*3.0; 0.26*-*3.0)***	**4.10; 4.10** ***(NR: 0.26*-*3.0; 0.26*-*3.0)***	**2.60; 2.60** ***(NR: 0.26*-*3.0; 0.26*-*3.0)***	**2.90; 2.90** ***(NR: 1.8*-*3.2; 1.8*-*3.2)***	**9.60; 9.60** ***(NR: 1.2*-*5.8; 1.2*-*5.8)***		**31.10; 31.10** ***(NR: 2.0*-*9.2; 2.0*-*9.2)***
**Testosterone (ng/dL; nmol/L)**	**<4; <0.14** ***(NR: <2.5*-*10; <0.09*-*0.35)***	**<4; <0.14** ***(NR: <2.5*-*10; <0.09*-*0.35)***	**<4; <0.14** ***(NR: <2.5*-*10; <0.09*-*0.35)***	**<4; <0.14** ***(NR: <2.5*-*10; <0.09*-*0.35)***	**50; 1.73** ***(NR:18*-*150; 0.63*-*5.20)***	**175; 6.07** ***(NR: 100*-*320; 3.47*-*11.09)***		**448; 15.53** ***(NR: 200*-*620; 6.93*-*21.50)***
**Inhibin B (pg/mL; ng/L)**		**19; 19** ***(NR: 35*-*167; 35*-*167)***	**37; 37** ***(NR: 35*-*167; 35*-*167)***					**8; 8** ***(NR: 74.0*-*470.0; 74.0*-*470.0)***
**AMH (ng/mL; pmol/L)**				**28.67; 204.79** ***(NR: 45.26*-*191.34; 323.29*-*1366.72)***				
**Stretched penile length, cm**	**2**	**3**		**3.5**	**4**		**7**	**7**
**Testicular size**					**1.5 cm**		**5cc; 5 mL**	**8 cc; 8 mL**

* The February 2021 visit was switched to telehealth and physical visit was postponed until August 2021 because of COVID-19.

** Normal ranges are noted in italics.

Abbreviations: AMH, antimüllerian hormone; FSH, follicle-stimulating hormone; LH, luteinizing hormone; NR, normal reference range.

Peripheral blood for 46,XY DSD next-generation sequencing (NGS) gonadal dysgenesis panel found a novel, dominant, heterozygous, likely pathogenic variant of the *NR5A1* gene at c102+ 1G > C. This is a canonical splice donor site of intron 2. This sequence change affects normal splicing of the *NR5A1* gene resulting in abnormal SF-1 protein with impaired function.

## Treatment

Therapy for the presumed diagnosis of CAH was stopped. The patient was referred to pediatric urology, which provided surgical and medical treatment over the next 4 years. He was started on a 6-month course of 50 mg/month intramuscular testosterone cypionate treatment to aid with penile growth and enhance vascular supply prior to hypospadias repair. He then underwent bilateral inguinal orchiopexy to position each testicle in its respective hemiscrotum.

Repair of his hypospadias subsequently involved a 2-stage preputial skin-flap repair. During the first stage, the substantial penile curvature was corrected and his shaft and preputial skin were deployed to the ventral aspect of the penis to provide tissue for the subsequent urethroplasty. The second stage was performed 7 months later during which the neourethra was created and the bifid scrotum was corrected. The neourethra measured 11 cm in length, sufficient to position the meatus at the top of the glans. At the most recent urology follow-up visit, cosmetic results were appropriate with no postoperative complications and he voided with a good stream.

## Outcome and Follow Up

By age 11 years, he had Tanner stage 3 pubic hair. Stretched penile length was 7 cm and scrotal testes were 5 cc bilaterally. Laboratory testing included a total testosterone level of 175 ng/dL (NR: 100-320 ng/dL); 6.07 nmol/L (NR: 3.47-11.09 nmol/L), LH level of 4.4 mIU/mL (NR: 0.2-5.0 mIU/mL); 4.4 IU/L (NR: 0.2-5.0 IU/L), and FSH level of 9.6 mIU/mL (NR: 1.2-5.8 mIU/mL); 9.6 IU/L (NR: 1.2-5.8 IU/L).

**Figure 1.**
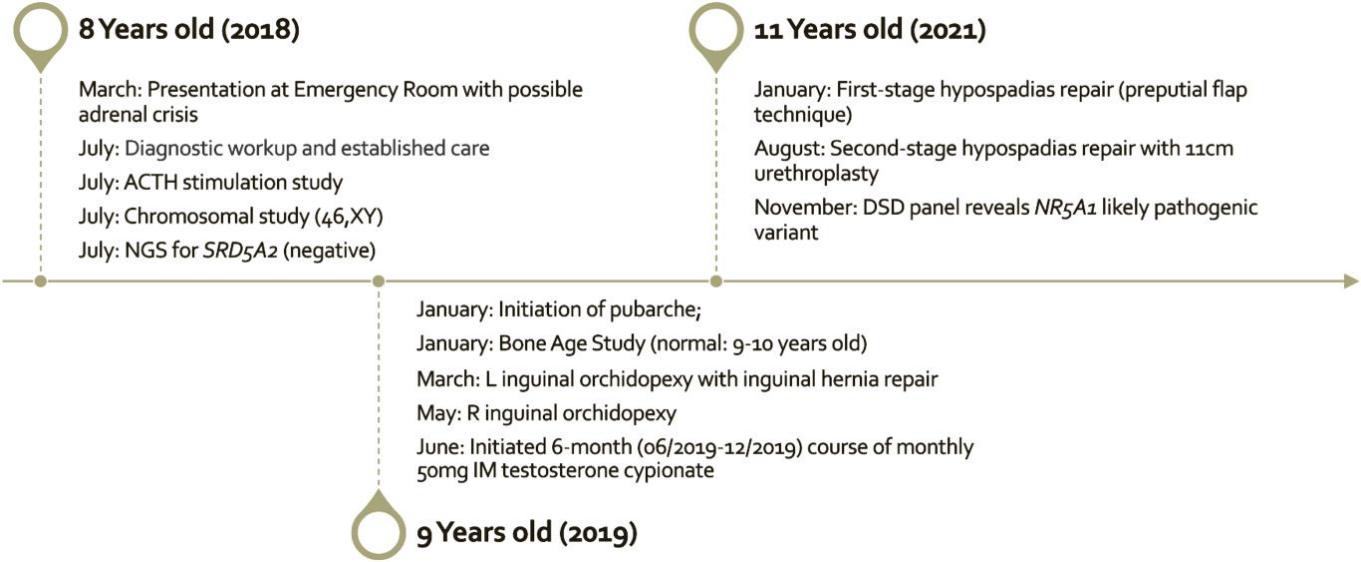
Timeline of significant medical, surgical, and procedural studies and results.

At the most recent visit, he was aged 13 years and has been growing in height (78th percentile) and weight (90th percentile); Tanner stage was 4 for pubic hair, stretched penile length was 7 cm, and bilateral testicular volume were 8 to 10 cc, minimally increased since the prior examination. Normal erections and nocturnal ejaculations were reported. Laboratory values showed a total testosterone level of 448 ng/dL (NR: 200-620 ng/dL); 15.5 nmol/L (NR: 6.93-21.50 nmol/L), LH of 25.00 mIU/mL (NR: 0.4-7.0 mIU/mL); 25.00 IU/L (NR: 0.4-7.0 IU/L), and FSH of 31.10 mIU/mL (NR: 2.0-9.2 mIU/mL); 31.10 IU/L (NR: 2.0-9.2 IU/L). He is not currently taking any endocrine medications; the clinical timeline is outlined in Table 1 and Fig. 1.

## Discussion

When this patient originally presented to the emergency department with fatigue and abdominal pain, he reported CAH, but had been without medical therapy for more than 3 months. Hence, there was concern for possible adrenal crisis. However, vital signs and electrolyte levels were normal. He was not hypoglycemic or hypercalcemic, denied nausea or vomiting, and did not exhibit signs or symptoms of adrenal crisis [7]. His undervirilization was not consistent with 21-hydroxylase CAH in a 46,XY individual, but may be consistent with other DSD/CAH diagnoses.

It is of interest that this individual, born in the Dominican Republic, was given an incorrect diagnosis initially with several possible reasons why this may have occurred. He was born in a country where CAH is not routinely checked on national neonatal screening tests. The patient had arrived in the United States without medical records, laboratory reports, or genetic testing documentation for CAH. It is unlikely that newborn screening for CAH and confirmatory genetic testing or NGS were completed or available. After specialized assessment at our center with the identification of a previously unknown variant of the *NR5A1* gene, he was treated surgically and has developed physically into midpuberty. Pubertal initiation was spontaneous and occurred 1 year prior to exogenous testosterone administration, which was given to optimize the hypospadias surgical repair. At his last visit, total testosterone remained normal although LH and FSH were elevated and values were concerning for future primary testicular failure.

**Figure 2.**
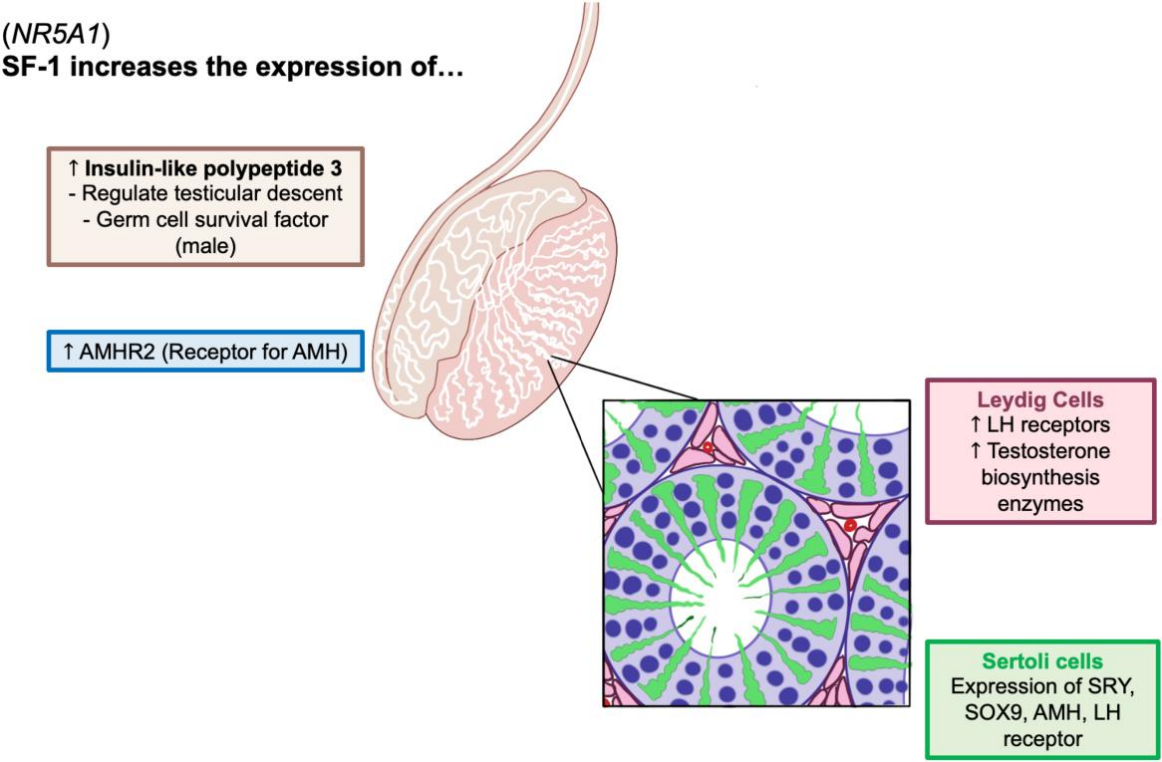
*NR5A1* mutation results in disrupted function of transcription factor steroidogenic factor-1 (SF-1). The novel, likely pathogenic variant in *NR5A1* is proposed to affect SF-1 function, resulting in this case presentation of 46,XY differences of sex development.

Cases of 46,XY DSD may present with a spectrum of ambiguous external genitalia without adrenal insufficiency [5]. Specifically, this patient presented with partial gonadal dysgenesis where a variant in the *NR5A1* disrupted the production of SF-1, a transcription factor found to activate essential sexual differentiation genes, such as the LH receptor (*LHCGR*), steroidogenic acute regulatory protein (*STAR*), cytochrome P450 family 11A1 (*CYP11A1*), and cytochrome P450 17A1 (*CYP17*) required for testosterone synthesis in Leydig cells. SF-1 has also been found to increase expression of insulin-like polypeptide 3, which regulates testicular descent, as well as the AMH receptor type 2 (*AMHR2*) required for normal male reproductive development (Fig. 2) [8]. Depending on the location of the variant, a wide range of genital presentations have been presented, including microphallus, hypospadias, clitoromegaly, and cryptorchidism. Other cases of 46,XY DSD with *NR5A1* mutations have reported severe gonadal dysgenesis with loss of androgens and AMH resulting in development of female external genitalia with clitoromegaly. Several individuals were raised as female because of phenotypic findings at birth, even though they all possessed testes without any residual müllerian structures [6]. In this case, the patient presented with ambiguous genitalia, hypospadias, chordee, bifid scrotum, and undescended testes, not unlike clinical presentations of patients cited in existing literature. Additionally, while a majority of cited *NR5A1* variants feature mutations in exons 3 or 4, this patient carried a novel mutation in a canonical splice donor site for intron 2. In the literature, it has been mentioned that those severely affected by gonadal dysgenesis may have increased risk of developing precursor germ cell tumors, including gonadoblastoma and seminoma. This may warrant early surveillance for malignancy [4].

Following the elimination of life-threatening diagnoses, patients with impalpable testes and hypospadias should be considered to have a DSD until proven otherwise. Of note, there is no consensus on the need for workup on those individuals with isolated hypospadias [9]. On evaluation, patients with possible DSD should start with karyotyping to allow for categorization of DSD type. This should be followed up with further assessment: serum electrolytes, testosterone, LH, FSH, and 17-OHP. Definitive diagnoses of enzyme deficiencies can usually be made based on the profile levels of hormones. Definitive genetic diagnosis requires sequencing through a karyotype-specific DSD variant panel, for example, the 46,XY panel focuses on *SRY, SOX9, SF1*, and other associated genes. Additionally, a copy number variant microarray may be used to determine microdeletions or duplications undetectable through NGS [10]. Newly available technology for the evaluation of rare disorders such as DSD has made a remarkable difference in their investigation, understanding, and treatment. Unfortunately, children born in countries without extended panels of neonatal screening and without advanced genetic testing may face challenges before receiving a precise diagnosis. Pediatricians taking care of patients immigrating from developing countries need to perform an exhaustive evaluation to determine the need for prompt referral to pediatric specialists for focused medical care and timely treatment.

## Learning Points

- Genetic evaluation has made a remarkable difference in the investigation and treatment of children with DSDs.
- Consider reevaluation in patients whose clinical presentation does not fit the original diagnosis, particularly those without full medical history records or who have recently immigrated from developing countries.
- 46,XY *NR5A1* patients can present with a broad phenotype including microphallus, hypospadias, clitoromegaly, and cryptorchidism.

## Data Availability

Original data generated and analyzed for this case report are included in this published article.

## Abbreviations

17-OHP: 17-hydroxyprogesterone
AMH: antimüllerian hormone
CAH: congenital adrenal hyperplasia
DSDs: differences of sex development
FSH: follicle-stimulating hormone
LH: luteinizing hormone
NGS: next-generation sequencing
NR: normal range
SF-1: steroidogenic factor-1
